# Calculated arterial blood gas values from a venous sample and pulse oximetry: Clinical validation

**DOI:** 10.1371/journal.pone.0215413

**Published:** 2019-04-12

**Authors:** Magnus Ekström, Anna Engblom, Adam Ilic, Nicholas Holthius, Peter Nordström, Ivar Vaara

**Affiliations:** 1 Department of Clinical Sciences, Division of Respiratory Medicine & Allergology, Lund University, Lund, Sweden; 2 Department of Medicine, Blekinge Hospital, Karlskrona, Sweden; 3 Department of Clinical Chemistry, Blekinge Hospital, Karlskrona, Sweden; University Magna Graecia of Catanzaro, ITALY

## Abstract

**Background:**

Arterial blood gases (ABG) are essential for assessment of patients with severe illness, but sampling is difficult in some settings and more painful than for peripheral venous blood gas (VBG). Venous to Arterial Conversion (v-TAC; OBIMedical ApS, Denmark) is a method to calculate ABG values from a VBG and pulse oximetry (SpO_2_). The aim was to validate v-TAC against ABG for measuring pH, carbon dioxide (pCO_2_) and oxygenation (pO_2_).

**Methods:**

Of 103 sample sets, 87 paired ABGs and VBGs with SpO_2_ from 46 inpatients eligible for ABG met strict sampling criteria. Agreement was evaluated using mean difference with 95% limits of agreement (LoA) and Bland-Altman plots.

**Results:**

v-TAC had very high agreement with ABG for pH (mean diff_(ABG–v-TAC)_ -0.001; 95% LoA -0.017 to 0.016), pCO_2_ (-0.14 kPa; 95% LoA -0.46 to 0.19) and moderate to high for pO_2_ (-0.28 kPa; 95% LoA -1.31 to 0.76). For detecting hypercapnia (PaCO_2_>6.0 kPa), v-TAC had sensitivity 100%, specificity 93.8% and accuracy 97%. The accuracy of v-TAC for detecting hypoxemia (PaO_2_<8.0 kPa) was comparable to that of pulse oximetry. Agreement with ABG was higher for v-TAC than for VBG for all analyses.

**Conclusion:**

Calculated arterial blood gases (v-TAC) from a venous sample and pulse oximetry were comparable to ABG values and may be useful for evaluation of blood gases in clinical settings. This could reduce the logistic burden of arterial sampling, facilitate improved screening and follow-up and reduce patient pain.

## Introduction

Arterial blood gases (ABG) are essential in everyday clinical care for evaluating acid-base status (pH), level of carbon dioxide (PaCO_2_) and oxygenation (PaO_2_) in patients with cardio-respiratory disease and severe illness. ABG is the current gold standard for assessing the need for acid-base correction, ventilator therapy and treatment with supplemental oxygen.[1–3]

ABG sampling is time consuming and might not be feasible in some settings as it needs to be performed by a physician or specially trained health care worker. This is especially problematic when blood gases need to be repeated to monitor the patient’s status over time. An indwelling arterial catheter is an option in these patients, but is used mostly in the intensive care unit (ICU) setting. Arterial sampling is more painful for the patient compared with puncture for a peripheral venous blood gas (VBG).[4, 5] Insufficient assessment of blood gases and excessive oxygen supply in patients with suspected chronic obstructive pulmonary disease (COPD) has been related to increased mortality.[2, 6] Due to the logistic burden and discomfort related to ABG sampling, alternative techniques to obtain arterial blood gas values are desired.

Current alternatives to ABG for assessing blood gases have important limitations. In a recent study in patients with an acute COPD exacerbation, VBG had high agreement with ABG for pH (mean ABG-VBG difference 0.03; 95% limits of agreement [LoA], -0.05 to 0.11) but agreement was lower for pCO_2_ (-0.75 kPa; 95% LoA, -2.91 to 1.41 kPa).[4] Although a venous pCO_2_ below 6.0 kPa had 100% sensitivity to exclude hypercapnia (PaCO_2_ < 6.0 kPa), specificity was only 57% and VBG was insufficient to evaluate the level of PaCO_2_ in the individual patient.[4, 7] VBG is also not useful for evaluating pO_2_.[2, 4, 7] Pulse oximetry is valuable for excluding hypoxemia but has limited specificity and precision for determining the presence of hypoxemia or its severity.[8] Pulse oximetry is therefore not recommended for evaluating the need for long-term oxygen therapy,[1] and gives no information concerning pH and pCO_2_.[2] Arterialized capillary gases from the earlobe can give measures of pH and pCO_2_ that are close to those of ABG and sampling is less painful.[2, 9] However, assessment of arterialized capillary gases requires standardized patient preparations, sampling and processing by specially trained staff and does not give accurate information on pO_2_.[2, 5, 9, 10]

v-TAC (Venous to Arterial Conversion Method) is a recent technique to obtain calculated ABG values based on a peripheral venous blood gas (VBG) and oximetry from a fingertip (SpO_2_).[11–14] The method has been evaluated by the developers in people with respiratory compromise including patients in emergency and intensive care, with reported v-TAC values of pH, pCO_2_ and pO_2_ similar to those of a concurrent ABG.[11–14] However, there has to date been no independent validation of v-TAC among inpatients with cardiopulmonary disease in clinical care.

The aim of the present study was to evaluate the agreement and clinical usefulness of v-TAC and VBG compared with ABG for the measurement of pH, pCO_2_ and pO_2_ among hospitalized patients eligible for blood gas assessment.

## Material and methods

### Design and eligibility

This was a cross-sectional comparison of v-TAC with ABG and VBG at the Department of Medicine, Blekinge Hospital, Karlskrona, Sweden. The study was approved by the Regional Ethics Committee of Lund University (Dnr: 2016/520). All participants provided written informed consent and the protocol the protocol is consistent with the principles of the Declaration of Helsinki. The paper is reported in accordance with recommendations for non-randomized trials [15] and comparing diagnostic tests.[16]

Patient inclusion criteria (all needed) were: age ≥ 18 years; hospitalized in the internal medicine and respiratory ward; had an indication for ABG assessment as judged by the responsible physician; SpO_2_ ≥ 75% in both hands; sufficient blood perfusion in both hands according to clinical status (adequate temperature, color, palpable radial pulse and normal reperfusion time [< 2 seconds]); possibility to take a peripheral antecubital venous sample and radial ABG in one of the arms as judged by the specialized study nurse; clinical stability before and during the sampling; and ability to provide informed written consent.

Exclusion criteria for blood samples were: time between ABG and VBG > 5 min; time between VBG and arrival to the laboratory > 15 min; time between VBG and analysis > 30 min; evidence of errors in sampling, processing and analysis such as visible gas bubbles, blood clots or unphysiological values for stable patients (arterial < venous for pH and pO_2_; arterial > venous for pCO_2_).

### Procedures and assessments

The study procedures were established and refined based on a pilot phase of 14 samples, which were not included in the main analysis. For the main study, patients admitted to the ward of pulmonary and internal medicine at the Department of Medicine, Karlskrona, Sweden were screened for eligibility by a specially trained nurse (AE) together with the responsible physician (including ME and AI) 2–3 days each week. Eligibility was confirmed in accordance with the inclusion and exclusion criteria and all participants obtained oral and written study information and provided written consent.

Assessments were performed and recorded by the trained study nurse (AE) in a standardized fashion. First, pulse oximetry was performed in both hands to confirm eligibility, and then continuously during the procedure on the non-test arm (to enable continuous measurement during venous sampling with tourniquet). SpO_2_ was measured using the same pulse oximeter (Rad-5v, Masimo, Neuchatel, Switzerland) for all patients for standardization and using finger probe as ear probe might be less reliable.[17] Second, an ABG was taken from the radial artery of the test arm using a standard arterial sample kit (Pro-Vent, Smiths Medical SD, Keene, USA) according to standard procedures. Third, an antecubital VBG was taken on the same test arm using a butterfly (BD Vacutainer, Becton Dickinson and Company, NJ, USA) and a blood gas sample kit (safePICO Aspirator, Radiometer Medical ApS, Brønshøj, Denmark). The samples were labeled with patient ID and assessment time and were not placed on ice. Data on patient and sample IDs, date and times of each assessment, test arm, and the mean SpO_2_ during the VBG puncture were recorded on a standardized study sheet.

The samples were not put on ice and were carried directly by hand to the Department of Clinical Chemistry situated in the same building. ABG and VBG values were analyzed using certified routine diagnostic methods on the instrument ABL800 FLEX (Radiometer Medical ApS, Denmark).

v-TAC values of pH, pCO_2_ and pO_2_ were calculated using the v-TAC software by OBI Medical based on the VBG values and concurrent SpO_2_. The v-TAC software by principle is a mathematical model of acid–base chemistry of blood, based on mass action and mass balance equations, including the effects of oxygen on the buffering characteristics of hemoglobin and the Bohr-Haldane effects, and a simulation algorithm that uses the mathematical model to simulate the mixing of venous blood with the same blood at elevated pO_2_ and reduced pCO_2_ levels until the calculated arterial saturation equals the measured SpO_2_. A detailed description of the method has been published elsewhere.[11, 12] The calculation of the v-TAC values was blinded to the ABG values. A patient could be sampled several times on separate days. Feasibility and validity of the procedures were evaluated in an interim analysis after ten collected samples.

Clinical characteristics were retrieved by one of the authors (AI) from the patients’ medical records regarding age, sex, primary cause of admission, comorbidities, date and values of latest spirometry and vital parameters (breathing frequency, blood pressure, pulse, and temperature) on the day of each test.

### Statistical analyses

Characteristics of patients and samples were tabulated as means with standard deviation (SD) and medians with range or interquartile range (IQR) for continuous variables with normal and skewed distribution, respectively. Categorical variables were expressed as frequencies and percentages. Correlations and comparisons were analyzed using mixed effects linear regression accounting for repeated assessments.

Mean difference in pH, pCO_2_ and pO_2_ was calculated for ABG–v-TAC and ABG–VBG, respectively. All comparisons were performed between the paired (concurrent) ABG and VBG. As v-TAC does not report pO_2_ values above 10 kPa these sample sets were not included in the analysis. Agreement of v-TAC and VBG with ABG was analyzed using Bland-Altman plots and 95% limits of agreement (LoA) were calculated.[18] As several assessments could be taken in the same patient on separate days, the LoAs were adjusted for repeated measurements to allow for clustering of values within each participants, using the method described by Bland and Altman (LoA = mean difference ± 2.77× standard deviation for samples within each participant).[18] Sensitivity, specificity, negative predictive value (NPV) and positive predictive value (PPV) were calculated for v-TAC and VBG and compared with ABG, for detecting hypercapnia (PaCO_2_ > 6.0 kPa), hypoxemia (PaO_2_ < 8.0 kPa) and severe hypoxemia (PaO_2_ < 7.4 kPa).

The analyses were performed on all included samples and for samples with SpO_2_ ≤ 88%, SpO_2_ ≤ 90% and SpO_2_ > 90%, respectively. To explore the influence of the sample eligibility criteria on the findings, a sensitivity analysis including all collected samples without visible air bubbles was conducted. Based on previous studies of v-TAC,[11–13] the sample size was pre-specified as 100 collected sample sets. Statistical significance was defined as two-sided p-value < 0.05. Statistical analyses were performed using the software package Stata version 14.2 (StataCorp LP; College Station, TX).

## Results

Between 21 Aug and 31 Oct 2017, a total 103 complete sample sets of consecutive ABG and VBG were collected. When evaluated against the quality criteria, 16 sets were excluded due to unphysiological values (n = 5), air bubbles in the syringe (n = 4), time between ABG and VBG > 5 min (n = 4), hyperventilation between the tests (n = 2) or SpO_2_ < 75% (n = 1).

The final analysis included 87 sample sets from 46 patients. Patient characteristics are shown in Table 1; mean age was 73.7 years, 61% were men, and the primary causes of admission were COPD exacerbation, heart disease and bacterial infection. The median time between the ABG and VBG was 3 (IQR 2–5) minutes, and the median time between VBG sampling and analysis was 9 (IQR 7–11) minutes (Table 1). The mean difference in saturation between ABG and pulse oximetry (SaO_2_–SpO_2_) was -0.6 (SD 2.6) percent points.

**Table 1:** Patient baseline characteristics.

Factor	Value
N	46
Age, mean (SD)	73.7 (12.7)
Males	28 (61%)
Primary cause of admission	
COPD exacerbation	13 (28%)
Heart disease	10 (22%)
Bacterial infection	8 (17%)
Cancer	2 (4%)
Hypoventilation	1 (2%)
Pulmonary fibrosis	1 (2%)
Pulmonary embolism	2 (4%)
Other	9 (20%)
Comorbidities	
Heart_disease	24 (52%)
COPD exacerbation	19 (41%)
Bacterial infection	10 (22%)
Cancer	10 (22%)
Hypoventilation	4 (9%)
Pulmonary fibrosis	2 (4%)
Pulmonary embolism	2 (4%)
FEV_1_, mean (SD)	1.13 (0.41)
FVC, mean (SD)	1.94 (0.60)
FEV_1_/FVC, mean (SD)	0.60 (0.18)
Number of samples per person	
1	26 (57%)
2	12 (26%)
3	4 (9%)
5	1 (2%)
6	3 (7%)
Minutes between arterial and venous sample, median (IQR)	3 (2–5)
Minutes between venous sample and analysis, median (IQR)	9 (7–11)

Data are presented as mean (standard deviation) or frequency (percent) unless otherwise stated.

*Abbreviations*: FEV_1_ = forced expired volume in one second; FVC = forced vital capacity; SD = standard deviation.

**Fig 1:**
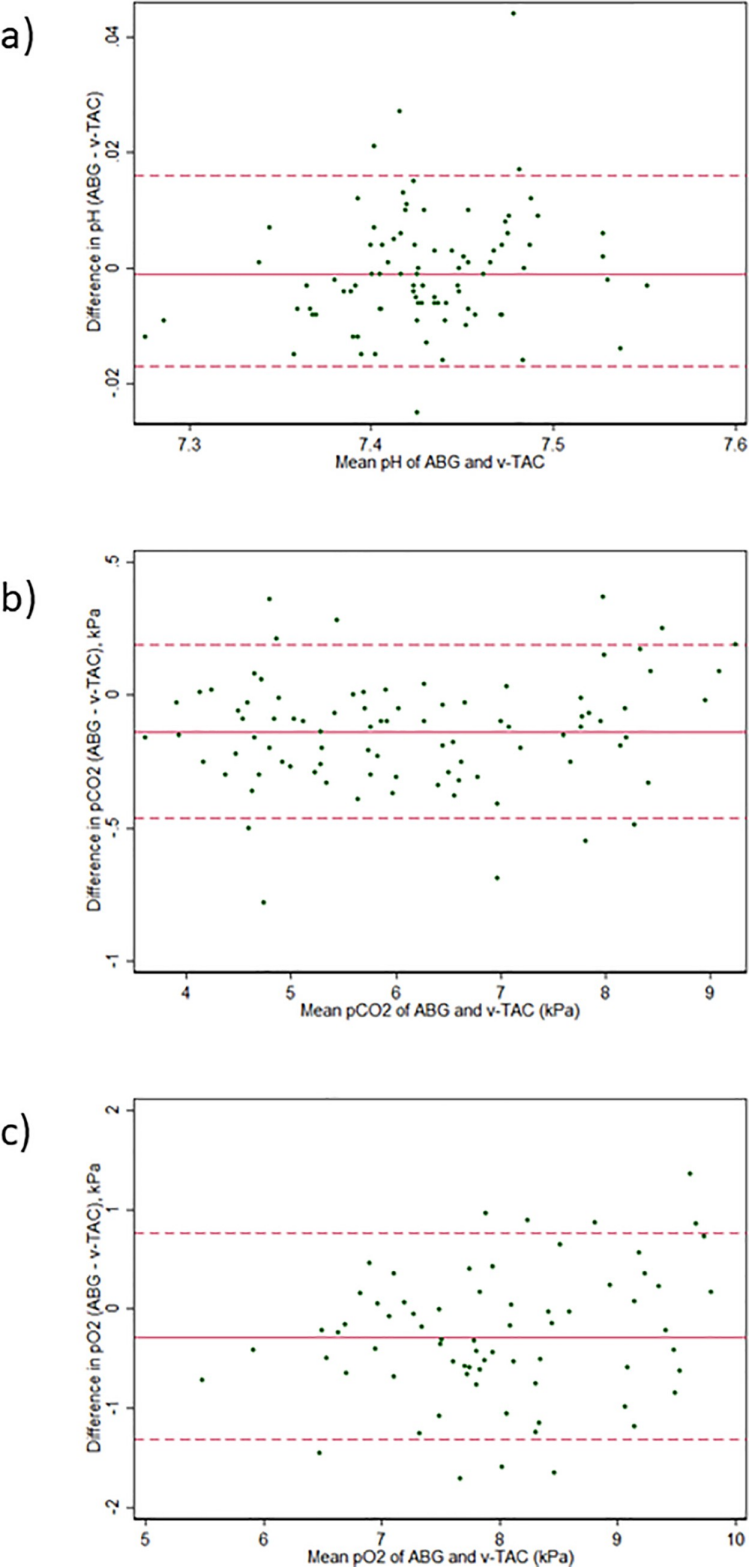
Bland Altman plots of agreement. Mean difference (solid line) with 95% limits of agreement (dashed lines), adjusted for repeated assessments (87 measurements in 46 patients). Agreement is shown for v-TAC vs. ABG regarding a) pH; b) pCO_2_; c) pO_2_.

Mean differences between ABG and v-TAC or VBG, respectively, are shown in S1 Fig in the online supplement. Compared with VBG, v-TAC yielded values closer to those of ABG for both pH, pCO_2_ and pO_2_. Bland-Altman plots are shown in Fig 1. Agreement was high between v-TAC and ABG for pH (mean diff_(ABG–v-TAC)_ -0.001; 95% LoA -0.017 to 0.016) and pCO_2_ (-0.14 kPa; 95% LoA -0.46 to 0.19), and moderate for pO_2_ (-0.28; 95% LoA -1.31 to 0.76), Table 2. Agreement was higher for v-TAC than for VBG for all analyses. The findings were similar between samples with SpO_2_ ≤ 90% and SpO_2_ > 90% (Table 2). v-TAC and ABG values were strongly correlated for pH (r = 0.98), pCO_2_ (r = 0.99) and moderately for pO_2_ (r = 0.81), p < 0.001 for all correlations.

**Table 2:** Comparison of pH, pCO_2_ and pO_2_ of v-TAC and venous blood gas compared with arterial blood gas.

Factor	All samples	Samples with SpO_2_≤90%	Samples with SpO_2_>90%	P-value between SpO_2_ groups *
Minutes between ABG and VBG, median (IQR)	3 (2, 5)	3 (2, 4)	3 (2, 5)	0.27
**pH**				
N samples	87	34	53	
ABG, mean (SD)	7.43 (0.05)	7.42 (0.05)	7.43 (0.05)	0.65
v-TAC, mean (SD)	7.43 (0.05)	7.42 (0.05)	7.43 (0.05)	0.31
Difference ABG–v-TAC, mean (SD)	-0.001 (0.010)	-0.004 (0.009)	-0.0001 (0.011)	0.50
95% limits of agreement	-0.017 to 0.016	-0.015 to 0.014	-0.015 to 0.013	
VBG, mean (SD)	7.41 (0.05)	7.41 (0.06)	7.41 (0.05)	0.97
Difference ABG-VBG, mean (SD)	0.021 (0.023)	0.016 (0.023)	0.025 (0.023)	0.087
95% limits of agreement	-0.018 to 0.060	-0.012 to 0.043	-0.010 to 0.059	
**pCO**_**2**_				
N samples	87	34	53	
ABG, mean (SD)	6.08 (1.45)	6.76 (1.41)	5.64 (1.30)	0.130
v-TAC, mean (SD)	6.21 (1.42)	6.89 (1.39)	5.78 (1.27)	0.043
Difference ABG–v-TAC, mean (SD)	-0.14 (0.21)	-0.13 (0.20)	-0.14 (0.21)	0.97
95% limits of agreement	-0.46 to 0.19	-0.44 to 0.18	-0.41 to 0.13	
VBG, mean (SD)	6.78 (1.58)	7.34 (1.56)	6.42 (1.49)	0.39
Difference ABG–VBG, mean (SD)	-0.70 (0.60)	-0.58 (0.62)	-0.79 (0.58)	0.158
95% limits of agreement	-1.66 to 0.25	-1.32 to 0.16	-1.61 to -0.04	
**pO**_**2**_				
N samples	70	34	36	
ABG, mean (SD)	7.84 (1.11)	7.06 (0.73)	8.93 (1.03)	<0.001
v-TAC, mean (SD)	8.12 (0.96)	7.37 (0.63)	8.83 (0.63)	<0.001
Difference ABG–v-TAC, mean (SD)	-0.28 (0.65)	-0.31 (0.56)	-0.25 (0.74)	0.96
95% limits of agreement	-1.31 to 0.76	-1.19 to 0.57	-1.11 to 0.62	
VBG, mean (SD)	5.61 (1.52)	5.60 (1.29)	5.61 (1.66)	0.92
Difference ABG–VBG, mean (SD)	2.59 (1.89)	1.46 (1.41)	-3.32 (1.81)	<0.001
95% limits of agreement	-0.35 to 5.54	-0.022 to 3.15	0.69 to 5.94	

The 95% limits of agreement are adjusted for repeated measurements in the same patient.[18] The number of sample sets is lower for pO_2_ as v-TAC does not report values > 10 kPa.

* Means are compared between samples with SpO_2_ ≤ 90% and SpO_2_ > 90% using random effects regression which accounts for repeated measurements.

*Abbreviations*: ABG = arterial blood gas; IQR = interquartile range; pCO_2_ = partial pressure of carbon dioxide in blood; pO_2_ = partial pressure of oxygen in blood; SD = standard deviation; VBG = venous blood gas; SpO_2_ = oxygen saturation from pulse oximetry; v-TAC = calculated arterial blood gas values from VBG and pulse oximetry.

Sensitivity, specificity and predictive values for v-TAC, VBG and SpO_2_ as compared with ABG are shown in Table 3. v-TAC identified all cases of hypercapnia (PaCO_2_ > 6.0 kPa; sensitivity 100%), as did VBG, but had higher accuracy for hypercapnia (97% *vs*. 84% for VBG). v-TAC was highly specific for the presence of hypoxemia (PaO_2_ < 8.0 kPa) and severe hypoxemia (PaO_2_ < 7.4 kPa). Consistently, v-TAC identified all patients with severe hypoxemia (PPV 100%). The accuracy of v-TAC for detecting hypoxemia was similar to that of pulse oximetry. v-TAC was more accurate than VBG for all analyses (Table 2). All findings were similar when analyzing all available sample sets without air bubbles (n = 99).

**Table 3:** Sensitivity, specificity and predictive values of v-TAC, VBG and SpO_2_ for hypercapnia and hypoxemia compared with ABG.

	ABG	v-TAC	VBG	SpO_2_ ≤ 90%	SpO_2_ ≤ 88%
**Hypercapnia (PaCO**_**2**_ **> 6.0 kPa), N = 39 (44.8%)**					
Sensitivity	*ref*	100.0 (91.0–100.0)	100.0 (91.0–100.0)		
Specificity	*ref*	93.8 (82.8–98.7)	68.8 (53.7–81.3)		
Accuracy (AUC)	*ref*	97 (93–100)	84 (78–91)		
PPV	*ref*	92.9 (80.5–98.5)	72.2 (58.4–83.5)		
NPV	*ref*	100.0 (92.1–100.0)	100.0 (89.4–100.0)		
**Hypoxemia (PaO**_**2**_ **< 8.0 kPa), N = 43 (49.4%)**					
Sensitivity	*ref*	60.5 (44.4–75.0)	97.7 (87.7–99.9)	72.1 (56.3–84.7)	41.9 (27.0–57.9)
Specificity	*ref*	93.2 (81.3–98.6)	11.4 (3.8–24.6)	93.2 (81.3–98.6)	97.7 (88.0–99.9)
Accuracy (AUC)	*ref*	77 (69–85)	55 (49–60)	83 (75–90)	70 (62–78)
PPV	*ref*	89.7 (72.6–97.8)	51.9 (40.5–63.1)	91.2 (76.3–98.1)	94.7 (74.0–99.9)
NPV	*ref*	70.7 (57.3–81.9)	83.3 (35.9–99.6)	77.4 (63.8–87.7)	63.2 (50.7–74.6)
**Severe hypoxemia (PaO**_**2**_ **< 7.4 kPa), N = 25 (28.7%)**					
Sensitivity	*ref*	64.0 (42.5–82.0)	100.0 (86.3–100.0)	92.0 (74.0–99.0)	68.0 (46.5–85.1)
Specificity	*ref*	100.0 (94.2–100.0)	16.1 (8.0–27.7)	82.3 (70.5–90.8)	96.8 (88.8–99.6)
Accuracy (AUC)	*ref*	82 (72–92)	58 (53–63)	87 (80–94)	82 (73–92)
PPV	*ref*	100.0 (79.4–100.0)	32.5 (22.2–44.1)	67.6 (49.5–82.6)	89.5 (66.9–98.7)
NPV	*ref*	87.3 (77.3–94.0)	100.0 (69.2–100.0)	96.2 (87.0–99.5)	88.2 (78.1–94.8)

Data presented as percent (95% confidence interval). Values for acidosis could not be calculated due to few cases (stable patients).

*Abbreviatons*: AUC = area under the curve of receiver operating analysis; NPV = negative predictive value (% of negatives that are true negatives); PPV = positive predictive value (% of positives that are true positives); for others see Table 2.

## Discussion

The main findings are that v-TAC has very high agreement with an ABG for level of pH and level of pCO_2_ and moderate to high agreement for pO_2_. v-TAC was more accurate than VBG for all measures.

This is the first published evaluation of v-TAC performed independently from the software developers. The present findings pertain to stable inpatients in a general respiratory and internal medicine ward. The results are consistent with previous reports from the developers in respiratory inpatients and patients admitted from an emergency department [14] and intensive care unit.[13]

Strengths of the present study are that it includes a relevant target population of inpatients with a range of cardiopulmonary conditions eligible for blood gas measurement. Sampling was standardized and conducted by a dedicated specially trained research nurse. The main analysis included only samples that met strict quality criteria. There were no signs of selection bias due to the eligibility criteria, as findings were robust when analyzing all available samples. The analysis aligned to recent recommendations on the comparison of diagnostic tests.[16]

Several limitations should be noted. Consecutive ABGs were not performed, as to optimize feasibility and validity of the sampling procedures (as ABG sampling is time consuming) and to limit patient burden and pain. Repeatability was not calculated between samples performed in the same patient, as the repeated assessments were taken on separate days. The observed mean difference in pO_2_ between ABG and v-TAC in the present study was not seen in previous reports.[11, 13, 19] This difference may represent a random variation or may be due to the average time from sampling to analysis of approximately 10 minutes, during which diffusion or continued metabolism in blood cells in the sample could lower the pO_2_ preferentially in oxygenated arterial blood.[20–22] The range of arterial pH was quite narrow with too few cases to permit analysis of acidosis or alkalosis, as testing was performed in patients who were in stable clinical conditions during the sampling. Sampling during clinical stability is essential when evaluating the agreement between tests as any change in the patient’s status between samples could bias the comparison. v-TAC was previously reported to be accurate also in hemodynamically unstable patients including patients treated with supplemental oxygen and non-invasive ventilation.[13] Strict sample eligibility criteria were applied to optimize the evaluation’s validity of the present evaluation.

The present findings have several potential clinical implications. v-TAC might be useful for routine evaluation of acid-base status and blood gases in some clinical settings and could reduce the need of arterial sampling. For ruling out acidosis and hypercapnia, normal values on a VBG may be sufficient, but more than 30% of elevated pCO_2_ values on VBG were false positives (PPV 68.8%). In contrast, v-TAC was more accurate for level of pH and pCO_2_ with values practically identical to those of the concurrent ABG. Importantly, v-TAC was superior for level of pCO_2_ compared with correcting the VBG value using the mean ABG-VBG difference, due to variation in the VBG value for the individual. V-TAC was more accurate than VBG for all measures.

Hypoxemia can be reliably excluded using pulse oximetry and the accuracy for detecting the presence of hypoxemia was similar between v-TAC and pulse oximetry. For assessing the level of hypoxemia (pO_2_), v-TAC was less accurate than for pH and pCO_2_, but the agreement between v-TAC and ABG was actually similar to or better than that reported between two consecutive ABGs.[13, 23, 24] The agreement between two consecutive ABGs was recently evaluated by Mallat *et al*.[23] Comparing two ABGs directly after each other using an arterial line in 192 stable intensive care patients,[23] the limits of agreement for PaO_2_ between two ABGs was ±1.2 kPa (9.1 mmHg), which is similar to the agreement for pO_2_ between v-TAC and ABG in the present study. For the clinician, this has two important implications: firstly, PaO_2_ is less precise than pH and PaCO_2_ and varies in a clinically significant way between consecutive ABGs. Therefore, pO_2_ and the level of hypoxemia should be evaluated by repeated measurements. The minimum detectable difference in PaO_2_ is about 1.2 kPa,[23] which makes ABG problematic as a “gold standard” when evaluating the patient’s pO_2_ and when evaluating other diagnostic methods. Secondly, for determining pO_2_, the performance of v-TAC is similar to that of ABG but is associated with lower risk of haematoma and markedly less pain.[4] Thus, v-TAC may be useful for repeated measurement and follow-up of blood gases. Capillary blood gas (CBG) assessment does not provide any advantages compared to v-TAC for measuring pH, pCO_2_ or pO_2_.[5, 19] In terms of implementation, v-TAC is a stand-alone software application that works together with existing blood gas analyzers on the market.

Clinical situations where v-TAC may be less useful and where further data are needed include in patients with hemodynamical instability and decreased peripheral perfusion; rapidly changing clinical status; SpO_2_ < 75%; dark skin; and when a peripheral venous sample cannot be obtained. Accurate entry of the SpO_2_ is important mainly for v-TAC prediction of pO_2_ whereas pH and pCO_2_ are relative robust to inaccurate SpO_2_ values.[11–14] As in all blood gas assessment, standardized valid sampling and analysis procedures are key to minimize pre-analytical error.

## Supporting information

S1 Fig Mean difference for v-TAC and VBG compared to ABG for a) pH; b) pCO_2_; and c) pO_2_.
(PPTX)

## References

[1] Hardinge M, Annandale J, Bourne S, Cooper B, Evans A, Freeman D, et al British Thoracic Society guidelines for home oxygen use in adults. Thorax. 2015;70(Suppl 1):i1–i43. 10.1136/thoraxjnl-2015-206865 25870317

[2] Driscoll BR, Howard LS, Earis J, Mak V. BTS guideline for oxygen use in adults in healthcare and emergency settings. Thorax. 2017;72(Suppl 1):ii1 10.1136/thoraxjnl-2016-209729 28507176

[3] Davidson AC, Banham S, Elliott M, Kennedy D, Gelder C, Glossop A, et al BTS/ICS guideline for the ventilatory management of acute hypercapnic respiratory failure in adults. Thorax. 2016;71(Suppl 2):ii1. 26976648 10.1136/thoraxjnl-2015-208209

[4] McKeever TM, Hearson G, Housley G, Reynolds C, Kinnear W, Harrison TW, et al Using venous blood gas analysis in the assessment of COPD exacerbations: a prospective cohort study. Thorax. 2015. 10.1136/thoraxjnl-2015-207573 PMC4789825 26628461

[5] Magnet FS, Majorski DS, Callegari J, Schwarz SB, Schmoor C, Windisch W, et al Capillary PO(2) does not adequately reflect arterial PO(2) in hypoxemic COPD patients. Int J Chron Obstruct Pulmon Dis. 2017;12:2647–53. 10.2147/COPD.S140843 PMC5593412. 28919732 PMC5593412

[6] Austin MA, Wills KE, Blizzard L, Walters EH, Wood-Baker R. Effect of high flow oxygen on mortality in chronic obstructive pulmonary disease patients in prehospital setting: Randomised controlled trial. BMJ. 2010;(of Publication: 30 Oct 2010):341 (7779) (pp 927), 2010. 10.1136/bmj.c5462. PMC2957540 20959284

[7] Martin CM, Priestap F. Agreement between venous and arterial blood gas analysis of acid-base status in critical care and ward patients: a retrospective cohort study. Can J Anaesth. 2017;64(11):1138–43. Epub 2017/08/25. 10.1007/s12630-017-0951-8 . 28836153

[8] Thrush D, Hodges MR. Accuracy of pulse oximetry during hypoxemia. South Med J. 1994;87(4):518–21. Epub 1994/04/01. . 8153783 10.1097/00007611-199404000-00019

[9] Zavorsky GS, Cao J, Mayo NE, Gabbay R, Murias JM. Arterial versus capillary blood gases: a meta-analysis. Respir Physiol Neurobiol. 2007;155(3):268–79. Epub 2006/08/22. 10.1016/j.resp.2006.07.002 . 16919507

[10] Eaton T, Rudkin S, Garrett JE. The clinical utility of arterialized earlobe capillary blood in the assessment of patients for long-term oxygen therapy. Respir Med. 2001;95(8):655–60. Epub 2001/09/04. 10.1053/rmed.2001.1118 . 11530953

[11] Rees SE, Hansen A, Toftegaard M, Pedersen J, Kristensen SR, Harving H. Converting venous acid-base and oxygen status to arterial in patients with lung disease. Eur Respir J. 2009;33(5):1141–7. Epub 2009/01/09. 10.1183/09031936.00140408 . 19129271

[12] Rees SE, Toftegaard M, Andreassen S. A method for calculation of arterial acid-base and blood gas status from measurements in the peripheral venous blood. Comput Methods Programs Biomed. 2006;81(1):18–25. Epub 2005/11/24. 10.1016/j.cmpb.2005.10.003 . 16303205

[13] Toftegaard M, Rees SE, Andreassen S. Evaluation of a method for converting venous values of acid-base and oxygenation status to arterial values. Emerg Med J. 2009;26(4):268–72. Epub 2009/03/25. 10.1136/emj.2007.052571 . 19307387

[14] Tygesen G, Matzen H, Gronkjaer K, Uhrenfeldt L, Andreassen S, Gaardboe O, et al Mathematical arterialization of venous blood in emergency medicine patients. Eur J Emerg Med. 2012;19(6):363–72. Epub 2011/11/16. 10.1097/MEJ.0b013e32834de4c6 . 22082876

[15] Lederer DJ, Bell SC, Branson RD, Chalmers JD, Marshall R, Maslove DM, et al Control of Confounding and Reporting of Results in Causal Inference Studies: Guidance for Authors from Editors of Respiratory, Sleep, and Critical Care Journals. Ann Am Thorac Soc. 2018 Epub 2018/09/20. 10.1513/AnnalsATS.201808-564PS . 30230362

[16] Mallett S, Halligan S, Thompson M, Collins GS, Altman DG. Interpreting diagnostic accuracy studies for patient care. BMJ: British Medical Journal. 2012;345. 10.1136/bmj.e3999 22750423

[17] Tittle M, Flynn MB. Correlation of pulse oximetry and co-oximetry. Dimensions of critical care nursing: DCCN. 1997;16(2):88–95. . 9104146 10.1097/00003465-199703000-00004

[18] Bland JM, Altman DG. Measuring agreement in method comparison studies. Stat Methods Med Res. 1999;8(2):135–60. Epub 1999/09/29. 10.1177/096228029900800204 . 10501650

[19] Klein AC, Rittger H. Validity and clinical use of mathematical arterialized venous blood gas with the v-TAC approach for evaluation of arterial blood gas in patients with respiratory compromise. Pneumologie. 2018;72(S 01):P342 10.1055/s-0037-1619175

[20] Beaulieu M, Lapointe Y, Vinet B. Stability of PO2, PCO2, and pH in fresh blood samples stored in a plastic syringe with low heparin in relation to various blood-gas and hematological parameters. Clin Biochem. 1999;32(2):101–7. Epub 1999/04/22. . 10211625 10.1016/s0009-9120(98)00098-8

[21] Knowles TP, Mullin RA, Hunter JA, Douce FH. Effects of syringe material, sample storage time, and temperature on blood gases and oxygen saturation in arterialized human blood samples. Respir Care. 2006;51(7):732–6. Epub 2006/06/28. . 16800906

[22] Srisan P, Udomsri T, Jetanachai P, Lochindarat S, Kanjanapattanakul W. Effects of temperature and time delay on arterial blood gas and electrolyte measurements. J Med Assoc Thai. 2011;94 Suppl 3:S9–14. Epub 2011/11/03. . 22043748

[23] Mallat J, Lazkani A, Lemyze M, Pepy F, Meddour M, Gasan G, et al Repeatability of blood gas parameters, PCO2 gap, and PCO2 gap to arterial-to-venous oxygen content difference in critically ill adult patients. Medicine (Baltimore). 2015;94(3):e415 Epub 2015/01/27. 10.1097/md.0000000000000415 25621691 PMC4602629

[24] Ladegaard‐Pedersen HJ. Accuracy and Reproducibility of Arterial Blood‐Gas and pH Measurements. Acta Anaesthesiol Scand. 1978;22(s67):63–5. 10.1111/j.1399-6576.1978.tb01375.x 28631

