# Supplementary figures and images for: Calculated arterial blood gas values from a venous sample and pulse oximetry: Clinical validation
Source: PLoS One. 2019 Apr 12;14(4):e0215413. doi: 10.1371/journal.pone.0215413 (PMC6461265; doi:10.1371/journal.pone.0215413)

## Slide 1
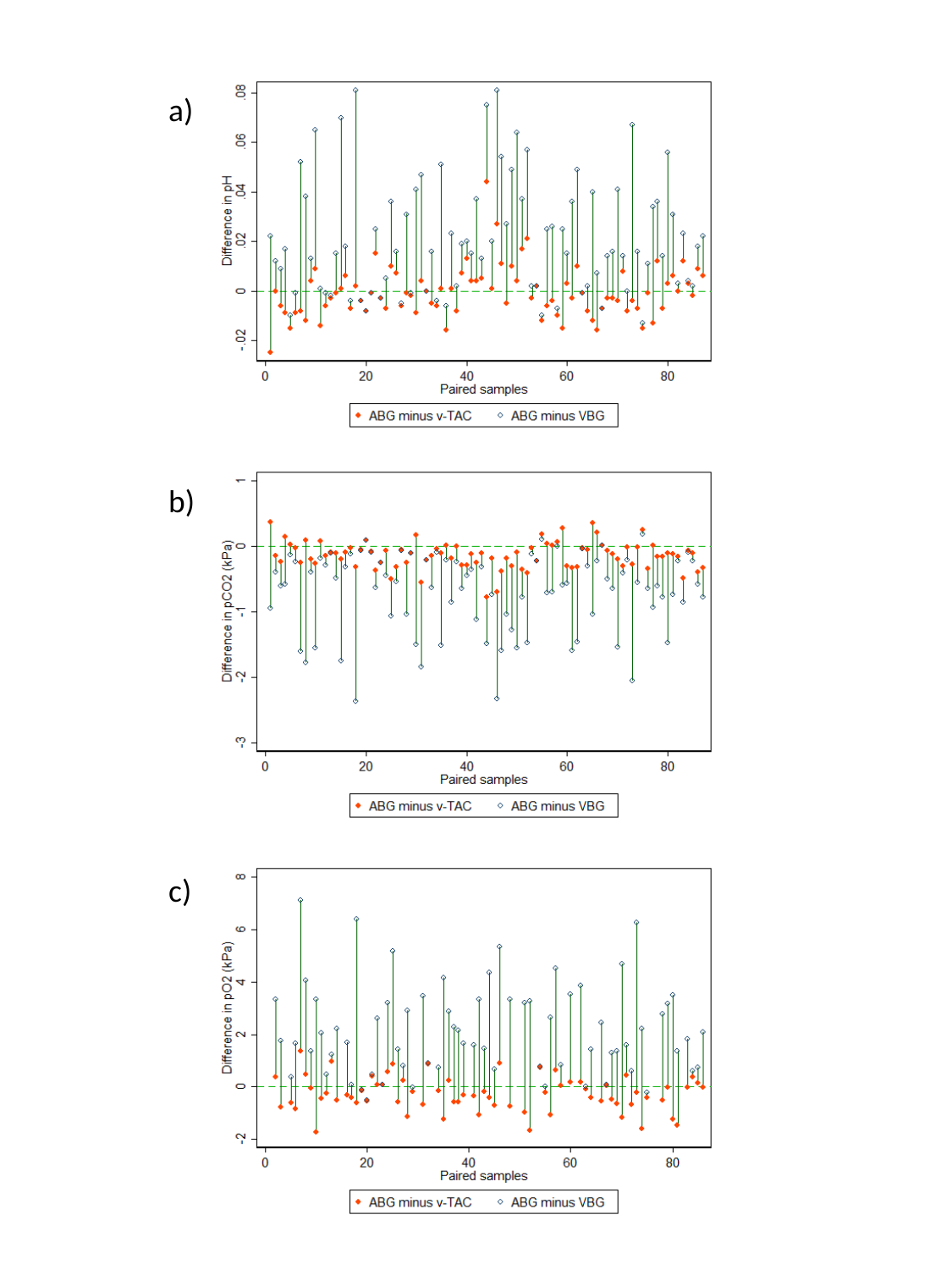

a)
b)
c)

Supplement: S1 Fig — Mean difference for v-TAC and VBG compared to ABG for a) pH; b) pCO2; and c) pO2. (PPTX) [file pone.0215413.s001.pptx]
